# Exercise therapy in the treatment of anorexia nervosa: Its effects depending on the type of physical exercise—A systematic review

**DOI:** 10.3389/fpsyt.2022.939856

**Published:** 2022-10-19

**Authors:** Marc Toutain, Antoine Gauthier, Pascale Leconte

**Affiliations:** UNICAEN, INSERM, COMETE, GIP CYCERON, Normandie University, Caen, France

**Keywords:** anorexia nervosa, eating disorders, exercise therapy, psychiatry, mental Health, physical exercise

## Abstract

**Background and purpose:**

Clinical research focusing on the effectiveness of exercise therapy (ET) in patients with anorexia nervosa (AN) shows increasing interest in the last decade. The aim of this systematic review was to provide an overview of quantitative studies that have examined the impact of ET in AN patients and to examine its specific effects on physical and mental health according to the type of physical exercise (PE) practiced.

**Methods:**

The review was carried out based on the PRISMA 2020. Electronic databases PubMed, Web of Science, Embase, and Wiley were searched from inception to December 2021. Quantitative studies assessing the effects of ET interventions on AN patients were included and study quality was assessed using the PEDro scale.

**Results:**

A total of 27 studies were selected, including 13 randomized controlled trials. Regarding outcomes measured, results showed that aerobic and resistance exercise improved muscle strength, that mind-body PE decreased main symptoms of AN and mental health, and that combined PE reduced dysfunctional exercise and improved weight gain.

**Conclusion:**

The findings suggest that ET intervention can induce benefits and has no deleterious effects on patients. In addition, specific effects on anorexia symptoms and physical and mental health have been observed according to the type of PE. However, this review reported several methodological weaknesses, including a lack of control group or randomization and statistical misconduct. Finally, ET intervention parameters were heterogeneous, and ET intervention generally lacked details, making reproducibility and comparability difficult. All these limitations underscore the need for a more rigorous methodology for further research.

## Introduction

Anorexia nervosa (AN) is an eating disorder (ED) that mainly affects women, particularly teenagers aged 15 to 19 years, with a peak in frequency at age 16 (1, 2). According to a recent review, the lifetime prevalence of AN is 4% in females and 0.3% in males (3). This disease has a low recovery rate of 50% six years after initial hospitalization (4) and a high relapse rate of over 50% (5). Moreover, a review of nearly fifty years of research has confirmed that AN has the highest mortality rate of any mental disorder (6). The main symptoms of AN are strict and voluntary food deprivation over a long period of time, ranging from several months to several years and resulting in significant weight loss, increased fear of gaining weight, and a distorted perception of the body (7). AN is associated with other mental health complications, such as body image disturbances (8), mood disorders, low self-esteem, exercise dependence, cognitive impairments (2, 9, 10), or sleep disturbances (11). AN also induces serious physical consequences, largely related to undernutrition, such as stunted growth, bone fragility (2), decrease in muscular strength and muscular endurance, hormonal and metabolic disorders (10), as well as hair loss, and kidney and bowel problems (12).

To prevent the aforementioned complications and to heal patients most efficiently and sustainably, a multidisciplinary approach to the treatment of AN is recommended by public health authorities, such as the “Haute Autorité de Santé” in France (13). Thereby, the standard care is composed of somatic and refeeding monitoring, nutritional rehabilitation, psychosocial interventions (family group psychotherapy, cognitive behavioral therapy, etc.) and medication (anxiolytics, antidepressants) (12, 14–16). Nevertheless, recovery remains long and difficult due not only to the diversity and severity of the symptoms and the associated comorbidities but also because of the patients’ denial of the disease and their lack of adherence to the care program (2, 13, 17). To improve treatment efficacy and patient compliance, other therapies are usually included in the standard care, such as arts therapy, exercise therapy (ET), relaxation, massages, acupuncture, etc. (17, 18). Of these, ET is receiving increasing interest from dedicated units for ED. ET can be defined as physical exercises (PE) formally supervised by an exercise professional, in order to restore optimal mental and physical functioning for specific therapeutic goals (19). ET has already been integrated into the treatment of mental illnesses and is now considered a compelling therapy for ED in some hospital departments (20, 21).

However, in many countries, PE remains restricted in specialized units for ED because of the high occurrence of dysfunctional exercise in AN which is considered a common comorbidity among these patients (22–24). According to Rizk et al. (25), the prevalence of dysfunctional exercise varied considerably from 5% to 54% in patients with AN, depending on the number of criteria used for its definition (26). Since 1995, various studies have attempted to define dysfunctional exercise and have shown that it comprises two primary dimensions: a quantitative dimension and a qualitative dimension (23, 26–28). The quantitative dimension refers to the duration and intensity of the exercise. Several authors have suggested that exercise is dysfunctional when the weekly exercise duration is 6 hours or more (25, 28–30). However, there is no formal consensus on this criterion which is based on subjective assessments and observations (25, 27). The qualitative dimension refers to the compulsive and obsessive components of physical exercise, which are reflected in rigid exercise schedules, prioritization of exercise over other activities, episodes of exercise compulsion, and guilt and anxiety when sessions are incomplete or missed (25, 27). Thus, patients with AN regularly engage in dysfunctional exercise, especially in their room or out of sight, to increase weight loss (2, 9, 31). Even if physical activity is restricted or prohibited by the medical team, patients may continue to over exercise. The primary problem is that this dysfunctional exercise interferes with weight gain and the recovery process by increasing the body’s energy expenditure (32). It is often associated with poorer treatment outcomes, longer inpatient stays, and a higher risk of relapse and disease chronicity (27). In light of this, it has become clear that PE should not be prohibited in the care of patients with AN, but rather be supervised by a professional to manage and encourage healthy behavior during exercise, and thus contribute to reduced dysfunctional exercise (32, 33).

Over the last two decades, some ET programs have been developed within specialized units for ED to promote better adherence to treatment and achieve more effective and sustainable reductions in the main symptoms of AN and associated disorders (17, 32, 34). Even if this remains a minority and no official recommendations exist, the development of ET in standard care is a growing phenomenon (32, 35). Achamrah et al.’s (32) review and Bratland-Sanda’s et al. (34) publication both reported that different types of ET, including aerobic exercise, resistance exercise, and mind–body PE (MBPE), have been implemented for patients with AN and other ED. Aerobic exercise of low to moderate intensity involves sustained, continuous, or intermittent effort over time (e.g. walking, running, cycling, swimming or shadow boxing) (32, 34). Regarding the symptomatology and comorbidities of AN, this type of PE has been shown to have positive effects on physical and socio-psychological health (e.g., mood, depression, well-being, anxiety, and group relations) (32, 34–36). Resistance exercise involves exerting effort against resistance that is induced either with equipment (e.g., dumbbells, elastic bands, or machines) or without equipment (i.e., body weight) to increase muscular strength or endurance. Particularly for patients with AN, this type of PE has been revealed to elicit positive effects on muscle mass and body weight, as well as on other parameters such as metabolic adaptations, neuroplasticity, mental health (e.g., anxiety, depression, and behavioral changes), and bone density (32, 36–38). MBPE, such as yoga, Pilates, stretching, tai chi, and qigong, has also been implemented in the care of AN (17, 32, 33, 36). This type of PE combines body movement, mental focus and controlled breathing which can improve strength, flexibility and balance, as well as relax the body and release psychological tension to achieve a state of well-being (32, 39).

Some studies have investigated the impact of ET interventions on AN patients and shown positive effects on the main symptoms of the disease, and physical and mental health, as well as better behavior toward the health care team (32, 35). However, to our knowledge, there are still few studies assessing the effects of ET interventions in patients with AN. This is partly due to the difficulty of conducting interventional studies in public health, but also to the fact that, as seen previously, ET has long been prohibited in the treatment of AN (34, 40, 41). This lack of studies was reported by reviews published in the last decade, which emphasized that more experimental studies were needed to explore the effects of ET interventions on AN (32, 33, 35, 42–44). This lack of proof was particularly highlighted in the most recent review conducted by Quiles Marcos et al. (35), which identified only twelve studies from 1970 to December 2019 (35). In addition, to our knowledge, none of these reviews sought to examine the effects of ET in patients with AN according to the type of PE implemented. Similarly, ET intervention parameters, such as session duration, exercise intensity, frequency, and period duration, were not generally highlighted or discussed. To date, the public health challenge is not only to demonstrate the health benefits of ET in patients with AN but also to determine which types of PE and which parameters might be recommended in a clinical or a research setting. This is one of the major interests of this literature review.

Therefore, the objectives of this systematic review are (i) to systematically review interventional studies that have assessed the effects of ET interventions in patients with AN, (ii) to examine effects according to the type of PE practiced on AN symptomatology and physical and mental health of patients, (iii) to examine the parameters of ET intervention and (iv) to discuss the relevance and limitations of these studies.

## Materials and methods

### Protocol and registration

This systematic review was carried out based on the 27 items of the PRISMA 2020 statement (Preferred Reporting Items for Systematic Reviews and Meta-Analyses) (45). The review was registered in the International Prospective Register of Systematic Reviews (PROSPERO): registration number CRD42022304532, available at https://www.crd.york.ac.uk/prospero/display_record.php?ID=CRD42022304532.

### Eligibility criteria

The eligibility criteria were formulated using the Population, Intervention, Comparison, Outcome, Study design (PICOS) framework, and two additional criteria were added: publication and date. We also added filters according to these criteria when applicable in databases (Table 1).

**TABLE 1:** PICOS (population, intervention, comparison and outcome), study design, publication and date criteria.

Items	Inclusion criteria	Exclusion criteria
Population	• Patient with a formal diagnosis of anorexia nervosa (DSM, CIM, clinical diagnosis).	• Prevention community sample. • Subject with only an history of AN
Intervention	• Exercise therapy including physical exercise.	• Exercise educational component only.
Comparison	• Pre versus post intervention with or without another group.	/
Outcomes	• Main symptoms of eating disorders with dedicated tools. • Physical and mental health parameters	• Qualitative outcomes only.
Study design	• Clinical trials. • Randomized controlled trials. • Non-randomized controlled trials. • Non-controlled trials. • Non-randomized trials.	• Case report. • Review articles. • Posters. • Conference paper. • Study protocols.
Publication	• Published in English or in French. • Published in a peer-reviewed journal. • Access to full text.	• Unpublished studies. Gray literature.
Date	• From inception to December 31 2021	/

**Population:** Studies conducted with patients (i.e., in- and outpatients) diagnosed with AN were included. Studies carried out in other ED were also selected if they included patients with AN. Other studies, such as community sample prevention with participants at risk of ED were not included.

**Intervention:** Studies that have examined the effects of ET interventions on the main symptoms of AN and/or associated disorders were included, even if ET was combined with other therapy. ET with only an educational component and no PE program was not eligible.

**Comparison:** Pre- versus post-intervention studies with or without a comparison group were eligible for review.

**Outcomes:** Only quantitative studies were included in this review, as it should be difficult to assume a level of generalizability between quantitative and qualitative outcomes. Outcomes were grouped into three categories and are presented below:

• Symptomatology of ED: questionnaires of ED symptoms.
• Physical health: height, weight, body mass index (BMI), percentage of body fat, fat body mass, lean body mass, skin fold, skeletal muscle mass, heart rate, muscle strength (peak torque and 6-repetition maximum), muscle size (circumference and area), endurance measures (endurance time, oxygen volume uptake at anaerobic threshold and peak oxygen volume uptake), motor tests (timed up and go test, timed up and down stairs test, visual task, tactile estimation task), time to vital sign stabilization, bio-markers from blood analysis (nutritional status, bone health status and endocrinal status), and psychological variables.
• Mental health: depression, anxiety, quality of life, self-esteem, body image, body attitudes, alexithymia, state of mind, emotional regulation, body awareness, positive and negative affect, self-objectification, health profile, physical activity level, behavior toward exercise, interoception accuracy, and expectations and experience of treatment.

**Study design:** Randomized controlled trials (RCT), as well as non-randomized (NRCT) and uncontrolled trials (UT), were included. Although the cornerstone of clinical intervention research is generally considered to be the RCT, in areas where patient numbers are limited or the evidence is conflicting, systematic reviews drawing on a variety of sources can bring together all available evidence on a specific topic (46). Reviews, case reports, conference papers, posters, study protocols and letters to the editor were excluded.

**Publication:** Papers in English or French were included. Only available full-text articles published in peer-reviewed journals referenced in official databases were included. Unpublished or not yet published studies and gray literature were excluded.

**Date:** No limit in the past was applied and studies published until December 31, 2021, were included.

### Information sources and search strategy

A systematic search of records was conducted by two authors (MT and PL) covering the period ranging from inception to December 31, 2021, in the following databases: PubMed, Web of Science, Embase (Elsevier) and Wiley Online Library. The search queries were designed according to Medical Subject Headings (MeSH) terms and usual key word terms according to the literature, combined with the Boolean operators “AND” and “OR” (Table 2).

**TABLE 2:** Databases search queries from inception to December 31 2021.

Database	Search query	Filter	Results
Web of Science	(TITLE = (’anorexia’ OR “eating disorder*” OR ‘anorexic*’) AND (’yoga’ OR ‘qigong’ OR ‘Pilates’ OR “physical activity” OR “physical therapy” OR “physical intervention” OR ‘dance’ OR ‘exercise’ OR ‘training’ OR “tai chi” OR ‘stretching’))	Articles	372
Embase	(’anorexia’:ti OR ‘eating disorder*’:ti OR ‘anorexic*’:ti) AND (’yoga’:ti OR ‘qigong’:ti OR ‘Pilates’:ti OR ‘physical activity’:ti OR ‘physical therapy’:ti OR ‘physical intervention’:ti OR ‘dance’:ti OR ‘exercise’:ti OR ‘training’:ti OR ‘tai chi’:ti OR ‘stretching’:ti)	Article + Article in press	346
PubMed	(“exercise therapy”[MeSH Terms] OR “exercise movement techniques”[MeSH Terms]) AND (“anorexia nervosa”[MeSH Terms]) OR ((anorexia[Title] OR anorexic*[Title] OR “eating disorder*”[Title]) AND (yoga[Title] OR qigong[Title] OR Pilates[Title] OR “physical activity”[Title] OR “physical therapy”[Title] OR “physical intervention”[Title] OR dance[Title] OR exercise[Title] OR training[Title] OR “tai chi”[Title] OR stretching[Title]))	Clinical trial Randomized controlled trial	51
Wiley	(’anorexia’ OR “eating disorder*” OR ‘anorexic’ OR “eating disorders” OR ‘anorexics’) AND (’yoga’ OR ‘qigong’ OR ‘Pilates’ OR “physical activity” OR ‘physical therapy’ OR ‘physical intervention’ OR ‘dance’ OR ‘exercise’ OR ‘training’ OR “tai chi” OR ‘stretching’)” in Title	Journals	132

**FIGURE 1:**
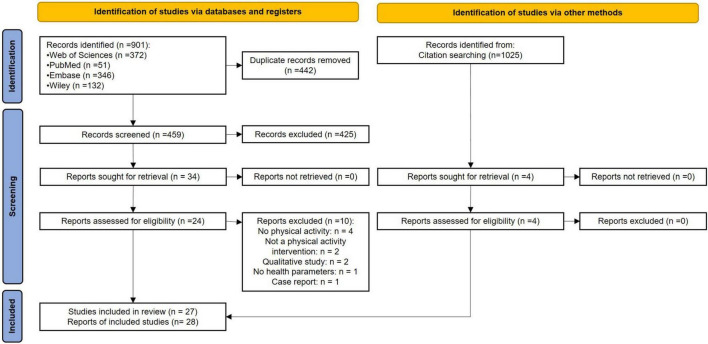
PRISMA flow diagram.

The selection diagram is reported in Figure 1. The author MT used the desktop Zotero software (version 5.0.96.3, Corporation for Digital Scholarship, Vienna, Virginia) to extract reports from databases, remove duplicates, and select reports for full-text eligibility inspection. The author PL extracted reports from databases in a word processing document, then removed duplicates, and selected reports for full-text eligibility inspection. After having removed duplicates, the two authors independently screened the titles and abstracts of the articles, and when necessary, the full text, to determine whether the inclusion or exclusion criteria were met. They then shared their results for the full-text articles included. After the full-text inclusion, the two authors independently reviewed the reference lists of each full-text article included to identify new eligible articles. In case of disagreement, a third author (AG) was consulted in the decision-making process.

### Data extraction

The following data were extracted from the selected articles: authors; year of publication; type of study; type of therapy; participant characteristics such as sample size, sex, age, BMI, ED type; attrition (post-intervention dropout, follow-up dropout); treatment settings (inpatient or outpatient); intervention parameters (type of PE, individualization status, instructor to patient ratio, session duration, exercise intensity, frequency and period); outcome measures; and significant results. This extraction included mean, standard deviation, and effect size when applicable. These data were first extracted in an Excel file and then transposed into four tables according to the type of PE provided within the ET (i.e., Table 3: aerobic exercise; Table 4: resistance exercise; Table 5: MBPEs; Table 6: combined PE).

### Quality assessment

The methodological quality of the reports selected was assessed using the Physiotherapy Evidence Database (PEDro) scale (47, 48), which is an 11-item scale designed to measure the internal validity and statistical quality of a clinical trial. Two reviewers (MT and PL) independently read and assessed the reports. In case of discrepancies, the two reviewers had to agree on a common score. Studies were rated on a scale of 0 to 10, with excellent quality scores of 9 or higher, good quality scores between 6 and 8, fair quality scores between 4 and 5, and poor quality scores between 0 and 3.

## Results

A total of 901 records were extracted from the 4 databases, from 1950 to December 31, 2021, and 442 duplicates were excluded. Then, 459 records were screened for eligibility based on title and abstract information, excluding 425 records that did not meet inclusion criteria. Of these, 34 reports were selected for full-text assessment; 24 reports met the eligibility criteria and 10 reports were excluded for the following reasons: 4 reports focused on other therapies (e.g., cognitive behavioral therapy or educational program); 2 reports focused on patients who exercised by themselves, 2 reports were qualitative studies, 1 report did not assess effects of ET intervention on health parameters and 1 report was a case report. The references of each article included were screened by two authors (MT and PL) in an Excel spreadsheet to identify other eligible articles allowing the inclusion of 4 additional reports. The references of these 4 added reports were also screened and no other article was included. In the end, 1,025 references were screened.

Among the 28 selected reports, we found seven reports corresponding to three studies (i.e., same sample, same trial number, same authors, same hospital): the two reports of Szabo & Green (49) and Chantler et al. (50) were from the same study; the three reports of Fernandez-del-Valle et al. (51), Fernandez-del-Valle et al. (52) and Fernandez-del-Valle et al. (53) were from the same study; and the two reports of Martinez-Sanchez et al. (54) & Martinez-Sanchez et al. (55) were from the same study.

According to the PRISMA 2020 guidelines, we pooled results together only for the two reports of Martinez and collaborators, since the experimental design and the participants were identical. In the end, 27 studies were included (Figure 1).

The 28 reports from the 27 studies included and examined in this review aimed to assess the effects of ET on ED symptoms and/or on the physical and/or mental health of patients with AN. Thirteen studies were RCTs (49–53, 56–63), 8 studies were NRCTs (8, 64–70), and 6 studies were UTs (54, 55, 71–75). All studies included patients with a diagnosis of AN; 11 studies also included patients with other ED as well (58–60, 62, 63, 66, 69, 70, 72–74); while 16 of the 27 studies included only patients with AN (8, 49–57, 61, 64, 65, 67, 68, 71, 75). Ten studies were conducted with inpatients (49, 50, 60, 62, 64, 66, 68, 69, 74, 75), 15 were conducted with outpatients (51–53, 56–59, 61, 63, 65, 67, 70–73) and 2 did not report the treatment setting (8, 54, 55). A total of 1,316 participants were included, consisting of 1,246 patients diagnosed with an ED, 715 of which had AN, and 70 healthy controls. Among all participants, 16 were males diagnosed with an ED, of which 5 were diagnosed with AN. Eight studies included only adolescents (51–56, 64, 72, 75), 8 studies included only adults (8, 59, 61, 63, 65, 67, 70, 71), 10 studies included both adolescents and adults (49, 50, 57, 58, 60, 62, 66, 68, 69, 74), and one study did not report the participants’ age (73). The mean age of all participants was 20.92 years, the lowest mean age was 12.61 years (51, 72), and the highest mean age was 36.1 years (61). Post-intervention, when reported, the overall mean dropout rate was 14.17% (± 15.10%), while the lowest dropout rate was 0% (56, 67), and the highest dropout rate was 45% (72). After the post-intervention follow-up period, when reported, the overall mean dropout rate was 10.34% (± 8.40%), the lowest dropout rate was 0% (63), and the highest dropout rate was 20% (62). In all studies, the PE sessions were supervised.

Regarding the aim of this systematic review, the 27 studies selected were divided into four categories according to the type of PE in the ET: (1) aerobic exercise (1 study) (64), (2) resistance exercise (7 studies) (49–53, 56, 57), (3) MBPE (11 studies) (8, 54, 55, 58–60, 65–67, 71–73), and (4) combined PE (8 studies) (61–63, 68–70, 74, 75).

### Effects of aerobic exercise on cardiorespiratory measures and body mass index

To our knowledge, the study of Tokumura et al. (64) is the only one to have assessed the effects of ET intervention based on aerobic exercise alone in patients with AN (64). The study protocol included 17 young inpatients with AN divided non-randomly into two groups, a control group following standard care and an ET group following standard care combined with a training program consisting of 30 minutes of stationary bicycling at participants’ individual anaerobic threshold level (approximately 50% of peak VO_2_) five times per week for 6 to 12 months (mean duration of 40 weeks). Compared to the control group, the intervention group demonstrated a significant increase in maximal oxygen uptake value and peak heart rate from pre- to post-program, as well as a significant increase in BMI (Table 3).

**TABLE 3:** Characteristics and main results of the study conducted on effects of aerobic exercise in patients with AN.

References	Study type	Type of therapy	Participants’ characteristics Sample size (% female), mean age (SD) Mean BMI (SD) and ED type (%)	Post-intervention dropout n (%)	Follow-up dropout, n (%)	Treatment settings	Intervention parameters		Outcome measures
		ET + SC	n = 9 (100%), 12 to 17 (NR)	NR	/				
			18.8 (± 0.5), AN (100%).				Stationary bicycle	D = 30 min	**Physical health:** BMI*; %BF; HR rest; HR peak*; Endurance time; VO2 at AT; Peak VO2*.
Tokumura et al. (64)	NRCT					Inpatient	Individualized: NR I/P ratio: NR NA = no	I = AT level F = 5x/week P = 6-12 months NA = no	
		SC	n = 8 (100%), 12 to 16 (NR) 19.6 (± 0.7), AN (100%)	NR	/				

SD, standard deviation; ED, eating disorders; ET, exercise therapy; BMI, body mass index; NRCT, non-randomized controlled trial; SC, standard care; AN, anorexia nervosa; NR, not reported; I/P ratio, Instructor/Patient ratio; D, session duration; I, intensity; F, frequency; P, period; NA, nutritional adjustment;%BF,% body fat, HR, heart rate; VO2 at AT, oxygen volume uptake at anaerobic threshold; peak-VO2, peak oxygen volume uptake; *, improvement for the intervention group.

### Effects of resistance exercise on muscular strength, body composition, bone remodeling and symptoms of AN

Seven RCTs investigated the beneficial effects of ET including only resistance exercises in patients with AN. These studies were conducted between 2002 and 2017. The resistance exercise programs were carried out two (Szabo and Green, (49); Chantler et al., (50); Fernandez-del-Valle et al., (56)), three (Fernandez-del-Valle et al., (51); (52); (53)) or fourteen times per week (Martin et al., (57)), with a session duration of 60 minutes, except for Martin et al.’s program (57) which included 5-minute sessions. Only one report did not mention the duration of sessions (Szabo and Green, (49)). ET intervention period lasted 8 weeks, except for a 3-month program by Fernandez-del-Valle et al. (56) and 9 days by Martin et al. (57). Exercise intensity, when documented, ranged from low to high (50–53, 56), and the studies by Fernandez-del-Valle et al. (51–53, 56) reported a progression in intensity. For example, in their 2010 study, the exercise intensity was 20–30% of 6-repetition maximum (6RM) at the beginning of the program and was progressed to 50–60% of 6RM by the end of the program (56). In their 2014 study, the exercise load was gradually increased from 70% of 6RM at the beginning of the program to 100% of 6RM at the end of the program (51). The summary of ET parameters from these reports highlighted a mean frequency of 4.14 (± 4.37) sessions per week, a mean session length of 50 minutes (± 22.13), and a mean ET period of 7.61 weeks (± 3.16).

Szabo and Green (49) and Chantler et al. (50), conducted an RCT with three comparative groups of seven participants (i.e., AN exercisers and AN non-exercisers from inpatient treatment settings and healthy community sample exercisers). In a first report, Szabo and Green (49) showed no intervention effect on body composition and psychological well-being and muscle strength after 8 weeks of resistance exercise with 2 sessions of 60 minutes per week (49). This ET intervention consisted of a series of exercises targeting a wide range of muscle groups (i.e., back, chest, thighs, hips, calves, shoulders, arms, abdominal) with 2.5kg dumbbells, elastic band and body weight. In a second report, Chantler et al. (50) conducted the same ET intervention (i.e., 60 min of light resistance training exercises twice a week for 8 weeks) and showed an increase in peak torque of knee flexors, knee extensors and elbow extensors for the AN exercisers group (Table 4) (50).

Two RCTs published by the same team in 4 reports (2010, 2014, 2015, and 2016) revealed congruent results in patients with AN. In their first study, Fernandez-del-Valle et al. (56) assessed the effect of a 3-month ET intervention including two weekly 60-min sessions of resistance exercises that varied from low-to-moderate intensity (56). The results showed a significant increase in upper body strength (51). In the other three reports from the same study, the authors examined the effect of 8 weeks of ET intervention including three weekly 60-minute sessions of resistance exercises, ranging from moderate to high intensity. The results surpassed those of the previous study and revealed significant increases in lower and upper body strength (51, 53), as well as in lower and upper body muscle mass, mid-thigh circumference and arm muscle area (52) (Table 4).

More recently, the study by Martin et al. (57) examined the effect of two daily sessions of twenty low jumps, performed over nine days, on weight gain, length of stay, stabilization of vital signs (rest heart rate and blood pressure) and, with an emphasis, on biological markers for bone remodeling in female patients with AN hospitalized for medical stabilization (57). Their results showed no significant difference, especially in bone remodeling biomarker concentrations. However, they revealed a shorter time to vital sign stabilization in the intervention group, as compared to the control group (Table 4).

**TABLE 4:** Characteristics and main results of studies (RCT, NRCT, UT) conducted on effects of resistance exercise in patients with AN.

Reference	Study type	Type of therapy	Participants’ characteristics Sample size (% female), mean age (SD) Mean BMI (SD) and ED type (%)	Post-intervention dropout, n (%)	Follow-up dropout, n (%)	Treatment settings	Intervention parameters		Outcomes
		ET + SC	*n* = 7 (100%), 20.1 (NR) 15.1 (± 1.1), AN (100%)	NR	/		Resistance exercise Individualized: NR I/P ratio: NR	D = NR I = N R F = 2x/week P = 8 weeks NA = yes	**Physical health:** BMI;%BF; FM; LM; EDI; BDI; Muscle strength (tool NR).
Szabo and Green (49)	RC T								
		ET (H)	*n* = 7 (100%), 21 (NR) 21.4 (± 2.7)	NR	/	Inpatient			
		
		SC	*n* = 7 (100%), 20.1 (NR) 16.5 (± 1.3), AN (100%)	NR	/				
									
		ET + SC	*n* = 7 (100%), 20 (± 5) 15.1 (± 1.1), AN (100%)	NR	/		Resistance exercise In dividualized: NR I/P ratio: NR	D = 60 min I = low F = 2x/week P = 8 weeks NA = yes	**Physical health:** B MI,%BF; FM; LM; Peak torque of knee extensors* and flexors*, elbow extensors* and flexors.
		
Chantler et al. (50)	RC T					Inpatient			
		
		ET (H)	*n* = 7 (100%), 23 (± 3) 21.4 (± 2.7)	NR	/				
		SC	*n* = 7 (100%), 22 (± 6) 16.5 (± 1.3), AN (100%)	NR	/				

		ET + SC	*n* = 11 (91%), 14.7 (± 0.6) 18.7 (± 1.7), AN (100%)	0 (0%)	/		Resistance exercise In dividualized: NR I/P ratio: 1/3	D = 60 min I = lo w to moderate F = 2x/week P = 3 months NA = no	**Physical health:** BF; LM; BMI; 6-RM SBP; 6-RM SLR*; 6-RM SLP; TUG-3m; TUG-10m; TUDS; SF-36.
Fernandez-del-Valle et al. (56)	RC T					Outpatient			
		SC	*n* = 11 (91%), 14.2 (± 1.2) 18.2 (± 1.5), AN (100%)	0 (0%)	/				

		ET + SC	*n* = 22 (100%), 12.61 (± 0.59) 17.28 (± 2.55), AN (100%)	0 (0%)	4 (18%)		Resistance exercise Individualized: NR I/P ratio: 1/2	D = 60 min I = hi gh F = 3x/week P = 8 weeks NA = yes	**Physical health:** BMI; 6-RM SBP*; 6-RM SLR*; 6-RM SLP*; TUG-3m; TUG-10m; TUDS.
Fernandez-del-Valle et al. (51)	RC T					Outpatient			
		SC	*n* = 22 (100%), 13 (± 0.6) 18.12 (± 2.11), AN (100%)	2 (9%)	2 (9%)				
		ET + SC	*n* = 22 (100%), 12.7 (± 0.7) 17.2 (± 2.4), AN (100%)	0 (0%)	/		Resistance ex ercise Individualized: NR I/P ratio: 1/2	D = 60 min I = moderate F = 3x/week P = 8 weeks NA = yes	**Physical he alth:** BMI; Triceps skinfold; mid-thigh skinfold; arm circumference; mid-thigh circumference*; arm muscle area* and thigh muscle area.
Fernandez-del-Valle et al. (52)	RC T					Outpatient			
		SC	*n* = 22 (100%), 13 (± 0.6) 18.3 (± 2.1), AN (100%)	2 (9%)	/				

		ET + SC	*n* = 22 (100%), 12.7 (± 0.7) 17.3 (NR), AN (100%)	4 (18%)			Resistance exercise Individualized: yes I/P ratio: NR	D = 50-60 m in I = moderate to high F = 3x/week P = 8 weeks NA = yes	**Physical heal th:** BMI;%BF; FM; SMM; Relative strength to body weight on 6-RM SBP*, 6-RM SLR* and 6-RM SLP*; Relative strength to LM on 6-RM SBP*, 6-RM SLR* and 6-RM SLP*; Skinfolds of biceps, triceps, subscapular and suprailiac; Circumference of thigh, arm and calf.
Fernandez-del-Valle et al. (53)	RC T					Outpatient			
		SC	*n* = 22 (100%), 13 (± 0.6) 18.1 (NR), AN (100%)	4 (18%)					

		ET + SC	*n* = 20 (95%), 16.8 (± 2.4) BMI = NR, AN (100%)	Global dropout n = 4/45			20 vertical jumps Individualized: NR I/P ratio: NR	D = 5 min I = NR F = 2x/day P = 9 days NA = no	**Physical he alth:** BMI; BSAP, NTX, OC and VSS*.
Martin et al. (57)	RC T					Outpatient			
		SC	*n* = 21 (95%), 16.8 (± 2.3) BMI = NR, AN (100%)	(9%)					

SD, standard deviation; ED, eating disorders; ET, exercise therapy; BMI, body mass index; RCT, randomized controlled trial; SC, standard care; H, healthy community sample; NR, not reported; AN, anorexia nervosa; I/P ratio, Instructor/Patient ratio; D, session duration; I, intensity; F, frequency; P, period; NA, nutritional adjustment; 6-RM, 6-repetition maximum; SBP, seated bench press; SLR, seated lateral row; SLP, seated leg-press; TUG-3m, timed up and go 3 minutes; TUG-10m, timed up and go 10 minutes; TUDS, timed up and down stairs;%BF,% body fat, FM, fat body mass; LM, lean body mass; SMM, skeletal muscle mass; EDI, eating disorder inventory; BDI, Beck depression inventory; SF-36,medical outcomes study 36-item short-form health survey; BSAP, bone-specific alkaline phosphatase; NTX, N-telopeptide; OC, osteocalcin; VSS, time to vital sign stabilization; *, significant improvement for the intervention group.

### Effects of mind-body physical exercise interventions

Eleven studies published in twelve reports investigated the benefits of MBPE interventions in patients with AN between 2008 and 2021, including three RCTs (58–60), four NRCTs (8, 65–67) and 4 UTs (54, 55, 71–73). Seven studies used yoga as ET intervention, one used basic body awareness therapy (i.e., based on massage and postural exercises), one used hoop training, one used Pilates, and one used dance movement therapy (Table 5). The MBPE interventions were carried out one (59, 66, 67, 71–73), two (39, 58), three (54, 55) or five times per week (60) depending on the study, with a session duration of 10 min (65), 60 min (8, 54, 58, 60), 75 min (67), 90 min (59, 66, 72) or 120 min (71). Only one study did not report the frequency of sessions (65). The duration of ET was 5 days (60), 8 weeks (58, 65, 66, 71, 73), 10 weeks (54, 55) or 12 weeks (8, 39, 72) depending on the study. Only one study carried out a single yoga session with patients with AN (67). The intensity of PE was not specified in all these studies. The summary of ET intervention parameters from these eleven studies highlighted a mean session length of 74.54 min (± 27.96), a mean frequency of 1.35 (± 0.74) sessions per week, and a mean ET duration of 8.5 weeks (± 4.56).

Of these studies, seven assessed the effects of ET on ED symptoms with validated questionnaires (55, 58–60, 67, 71, 72), such as the eating attitude test (EAT-26 and EAT-40) (76, 77), the eating disorder inventory (EDI versions 1, 2, and 3) (78–80), and the eating disorder examination questionnaire (EDE-Q) (81). Three studies showed a significant improvement in the ED symptoms for the intervention group post-program, and more specifically on the subscales assessing drive for thinness/body dissatisfaction and weight and shape concerns (59, 71, 72). Five studies assessed the effects on ED symptoms with other scales and four of them showed significant improvements in body image, body dissatisfaction and body attitude.

Seven studies evaluated the effect of ET interventions on psychological disorders associated with AN, such as anxiety, depression, positive and negative affect, self-esteem and quality of life. Of them, four studies revealed significant effects (Table 5). The results found by Hall et al. (72) showed that regular yoga training (i.e., one or two sessions per week for eight to twelve weeks) significantly decreased depression and anxiety scores, and improved state of mind in young women with AN or other ED (72). In their study, Pacanowski et al. (60) showed that one daily yoga session practiced for five days before dinner significantly reduced the negative affect of patients with AN or other ED, compared to the control group (60). Catalan-Matamoros et al. (59) revealed a significant increase in the mental health score assessed by the SF-36 quality of life questionnaire after twelve weeks of one weekly session of basic body awareness therapy (59, 82). In the same way, Martinez-Sanchez et al. (55) showed a significant improvement in quality of life on the Kid Screen-27 questionnaire for young anorexic patients after 10 weeks of Pilates (54).

The study by Martinez-Sanchez et al. (54) revealed additional significant results. They found an increase in plasma calcium, involved in various functions of the body, and a decrease in plasma follitropin, involved in ovum production. In addition, results showed improvements in sleep parameters, such as a decrease in duration and number of night perturbations and an increase in sleep efficiency (55).

Seven studies assessed the BMI of participants, but none revealed any effect of MBPE (54, 55, 58, 60, 66, 67, 71, 72). Four studies reported no significant effect on any of their measures (8, 58, 65, 67). The study by Keizer et al. did not perform any statistical analyses (65).

**TABLE 5:** Characteristics and main results of studies (RCT, NRCT, UT) conducted on effects of mind-body physical exercise in patients with AN.

References	Study type	Type of therapy	Participants’ characteristics Sample size (% female), mean age (SD) Mean BMI (SD) and ED type (%)	Post-intervention dropout, n (%)	Follow-up dropout, n (%)	Treatment settings	Intervention parameters		Outcomes
		ET + SC	*n* = 26 (92%), 10 to 21 (NR) 19.51 (± 3.01), AN (55%), BN (17%), EDNOS (28%)	2 (8%)	1 (4%)		Yoga Individualized: yes I/P ratio = 1/1	D = 60 min I = NR F = 2x/week P = 8 weeks NA = no	**ED symptoms:** EDE-Q; **Physical health:** BMI; **Mental health:** BDI-II; STAI.
Carei et al. (58)	RCT					Outpatient			
		SC	*n* = 27 (92%), 10 to 21 (NR) 18.88 (± 2.32), AN (55%), BN (17%), EDNOS (28%)	1 (4%)	1 (4%)				

		ET + SC	*n* = 14 (93%), 29,5 (NR) 18.8 (± 0.5), AN (36%), BN (50%), EDNOS (14%)	0 (0%)	/		BBAT (based on massages and postures exercises) Individualized: no I/P ratio = NR (in group)	D = 90 min I = NR F = 1x/week P = 12 weeks NA = no	**ED sympto ms:** EDI*; EAT-40*; **Mental health:** SF-36*; BAT*.
Catalan-Matamoros et al. (59)	RCT					Outpatient			
		SC	*n* = 14 (93%), 25,2 (NR) 19.6 (± 0.7), AN (37.5%), BN (37,5%), EDNOS (25%)	6 (43%)	/				

		ET + SC	*n* = 20 (100%), 26.8 (± 10.3) 21.5 (± 7.5), AN (58%), BN (21%), EDNOS (21%)	1 (5%)	/		Yoga before dinner Individualized: NR I/P ratio = NR	D = 60 min I = NR F = 1x/day P = 5 days NA = no	**ED symp toms:** EDE-Q. **Physical health:** BMI; **Mental health:** EAQ; PANAS*.
Pacanowski et al. (60)	RCT					Inpatient			
		SC	*n* = 18 (100%), 26.8 (± 8.7) 18 (± 3.9), BN (21%), EDNOS (21%)	1 (6%)	/				

		ET + SC	n = 19 (100%), 26 (± 6.5) 16.35 (± 2.49), AN (100%)	NR	/		Yoga/stretching Individualized: NR I/P ratio = NR	D = 60 min I = low F = 2x/week P = 3 months NA = no	**Mental health:** PSDQ; CDRS; RSES.
Moscone et al. (8)	NRCT					Inpatient			
		NI (H)	*n* = 16 (100%), 24.6 (± 3) 21.7 (± 2.99)	NR	/				

Keizer et al. (65)	NRCT	ET + SC	*n* = 16 (100%), 22.87 (± 2.9) 19.53 (± 1.04), AN (100%)	2 (12%)	/	Outpatient	Hoop training (moving in hoops according to body size perception) Individualized: yes I/P ratio = 1/1	D = 5-10 min I = NR F = NR P = 8 weeks NA = no	**Mental heal th:** BAT; VET; TET; HT.
		
		SC	*n* = 14 (100%), 23.17 (± 5.67) 20.11 (± 1.17), AN (100%)	2 (14%)	/				
		
		NI (H)	*n* = 20 (100%), 21.21 (± 1.44) 20.8 (± 1.61)	1 (5%)	/				

		ET + SC	*n* = 7 (100%), 20.1 (± 5.9) 19.82 (± 4.37), AN (43%), EDNOS (57%)	0 (0%)	/				
Savidaki et al. (66)	NRCT					Inpatient	Dance movement therapy Individualized: yes I/P ratio = 1/4-5	D = 90 min I = NR F = 1x/week P =4 to 11 sessions (mean = 7.71) over 14 weeks NA = no	**Physical health:** BMI; **Mental health:** TAS-20; MBSRQ*.
		SC	*n* = 7 (100%), 20.3 (± 2.5) 19.07 (± 2.28), AN (40%), BN (20%), EDNOS (40%)	2 (29%)	/				

		ET + SC	*n* = 15 (100%), 28 (± 11.22) 16.11 (± 4.33), AN (100%)	0 (0%)	/		One yoga class Individualized: yes I/P ratio = NR (in group)	D = 75 min I = NR F = 1x P = 1 day NA = no	**ED symptom s:** EDI-2. **Physical health:** BMI; **Mental health:** HAM-D; HAM-A; BAQ; TAS-20; SOQ; HR; Iac.
Demartini et al. (67)	NRCT					Outpatient			
		NI (H)	*n* = 20 (100%), 28.59 (± 9.85) 21.23 (± 3.12)	0 (0%)	/				

Cook-Cottone et al. (71)	UT	ET + SC	*n* = 29 (100%), 20 (NR) 18.3 to 29.3, AN (100%)	5 (17%)	/	Outpatientt	Yoga, relaxation and meditation Individualized: yes I/P ratio = 1/1 and NR for group sessions	D = 120 min I = NR F = 1x/week P = 6-8 weeks NA = no	**ED symptoms:** EDI-2. **Physical health:** BMI; **Mental health:** subscales: drive for thinness*, body dissatisfaction*, and bulimia;

Hall et al. (72)	UT	ET + SC	*n* = 20 (100%), 12.61 (± 0.59) 17.28 (± 2.55), AN (15%), BN (5%), ARFID (5%), OSFED (75%)	9 (45%)	/	Outpatient	Yoga Individualized: NR I/P ratio = NR	D = 60-90min I = NR F = 1x/week P = 12 weeks NA = no	**ED symptoms:** EAT-26*; EDE-Q*. **Physical health:** BMI; **Mental health:** SOM*; STAI*.

Diers et al. (73)	UT	ET + SC	*n* = 91 (99%), age = NR BMI = NR, AN, BN, OSFED (%NR)	24 (26%)	/	Outpatient	Yoga Individualized: yes I/P ratio = NR (in group)	D = 90 min I = NR F = 1x/week P = 8 weeks NA = no	**Mental health:** Body image questionnaire* (constructed by the first author).

Martinez-Sanchez et al. (54) and Martinez-Sanchez et al. (55)	UT	ET + SC	*n* = 15 (100%), 14.6 (± 1.7) 19.6 (± 2.2), AN (100%)	3 (20%)		NR	Pilates Individualized: yes I/P ratio = NR (in group)	D = 60 min I = NR F = 3x/week P = 10 weeks NA = no	**ED symptoms:** EDI-3. **Physical health:** BMI, impedance analysis for body composition (total body water, body FM, LM, SMM, FFM,%body fat, bone mineral content); 34 measures from blood analysis (calcium* and follitropin*); Sedentary time, night sleep duration*, sleep latency, night perturbations* and sleep efficiency*. **Mental health:** CDRS*; K27*;

SD, standard deviation; ED, eating disorders; ET, exercise therapy; BMI, body mass index; ED, eating disorder; RCT, randomized controlled trial; NRCT, non-randomized controlled trial; UT, uncontrolled trial; SC, standard care; NI, no intervention; H, healthy community sample; NR, not reported; AN, anorexia nervosa; BN, bulimia nervosa; EDNOS, eating disorder not otherwise specified; ARFID, avoidant/restrictive food intake disorder; OSFED, other specified feeding or eating disorder; I/P ratio, Instructor/Patient ratio; D, session duration; I, intensity; F, frequency; P, period; NA, nutritional adjustment; EDI-2, eating disorder inventory 2; EDE-Q, eating disorder examination questionnaire; BDI-II, Beck depression inventory-II; STAI, state and trait anxiety inventory; BBAT, basic body awareness therapy; EDI, eating disorder inventory; EAT-40, eating attitude test 40-items; SF-36,medical outcomes study 36-items short-form health survey; BAT, body attitude test; PSDQ, physical self-description questionnaire; CDRS, contour drawing rating scale; RSES, Rosenberg self-esteem scale; SOM, state of mind questionnaire; EAT-26, eating attitudes test 26-Item; EAQ, emotional avoidance questionnaire; PANAS, positive and negative affect schedule; VET, visual estimation task; TET, tactile estimation task; HT, hoop task; FM, fat mass; SMM, skeletal muscle mass; FFM, fat free mass;%BF,**%** body fat; EDI-3, eating disorder inventor-3; K27, KIDSCREEN 27; TAS-20, Toronto alexithymia scale, MBSRQ, multidimensional body-self relations questionnaire; HAM-D, Hamilton rating scale for depression; HAM-A, Hamilton anxiety rating scale; BAQ, body-awareness questionnaire; SOQ, self-objectification questionnaire; HR, heart rate; **Iac**, interoception accuracy; *, significant improvement for the intervention group.

### Effects of combined physical exercise interventions

Over the identified period, eight studies from 1993 to 2020 have investigated the effect of combined PE interventions (i.e., composed of at least 2 different types of PE) in patients with AN (Table 6) (61–63, 68–70, 74, 75). Of these studies, three were RCTs (61–63), three were NRCTs (68–70) and two were UTs (74, 75). The combined PE interventions were carried out one (75), two (62, 70, 74), three (61, 63) or four times per week (69) depending on the study, with a session duration of 60 min (69), 90 min (75), 100 min (62, 74), 120 min (63, 70), or 180 min (68) depending on the study. Only one study did not report the frequency of sessions (68), and only one study did not mention the duration of the session (61). The durations were 4 weeks (62, 74), 6 weeks (68), 8 weeks (75) or 12 weeks (61, 63, 70) depending on the study. Only one study did not report the duration of the ET intervention (69). The intensity was not documented in all of these studies. To manage progression, two studies reported adaptations to regulate PE intensity and difficulty (61, 75). The summary of ET interventions parameters from these eight studies highlighted a mean session length of 98.33 minutes (± 22.28), a mean frequency of 2.28 (± 0.95) sessions per week, and a mean duration of 8.14 weeks (± 3.84).

Touyz et al. (68) were the first to examine the effect of a combined PE intervention (i.e., 180 min per week for 6 weeks of stretching, posture enhancement, weight training, social sport, and occasional aerobic activity) on the health of patients with AN. They did not reveal any significant differences in BMI or weight gain after the intervention (68). In the same way, Thien et al. (61) showed no increase in BMI or body fat in patients with AN after a combined PE intervention including stretching, aerobic exercises and resistance training performed three times a week over three months. Nonetheless, their results revealed a trend in the improvement of quality of life in the intervention group compared to the control group (Table 6) (61).

Calogero and Pedrotty conducted the largest inpatient interventional study with 254 patients with ED. In this study, 127 patients underwent one month of ET intervention including four weekly sessions of 60 minutes of stretching, yoga, Pilates, strength training, balance and coordination practice and aerobic exercise, and were compared to 127 no-exercise patients (69). The results showed a significant increase in weekly weight gain, as well as total post-program weight regain in the intervention group compared to the control group. There was also a significant reduction in dysfunctional exercise (e.g., compulsiveness, physical hyperactivity), as assessed by questionnaire, for the intervention group (Table 6).

Schlegel et al. developed the Freiburg sport therapy program, designed for outpatients with ED (70). This program was conducted over 12 weeks with two 120-minute sessions per week. Each session was composed of an educational program focused on good practices and healthy behaviors concerning PE, coupled with team sports and body-oriented and playful PE. For the intervention group, the results showed a significant reduction in obligatory and excessive exercising assessed using the commitment to exercise scale (CES) (83). Based on this pilot study, Zeeck et al. conducted an outpatient RCT using the Freiburg sport therapy program (63). They found a significant reduction in unhealthy exercise behaviors assessed using the compulsive exercise test (CET) for the intervention group (84).

Two other studies obtained similar results with another ET intervention. The uncontrolled pilot study by Dittmer et al. assessed the effect of cognitive behavioral therapy (CBT) focused on changing practice behavior and attitude toward exercise, coupled with various PE (i.e., yoga, recreational activities, dual-task training, and body exploration) (74). After four weeks of two 100-min sessions per week, the results showed a significant increase in BMI, as well as a significant decrease in dysfunctional exercise behavior, desire for thinness, perfectionism, and depression level. Regarding this pilot study, Dittmer et al. carried out an RCT using the same protocol with a larger inpatient sample of 207 ED patients, including a majority of patients with AN (62). The results showed a significant reduction in compulsive exercise behavior measured using the CET and CES for the intervention group, after ET intervention and at the 6-month follow-up (Table 6).

The UT by Kern et al. examined the effects of one 90-minute session per week of resistance training and shadow boxing for 8 weeks (75). They found a significant increase in BMI and quality of life, as well as a significant decrease in exercise dependence, physical activity level, and specific symptoms of AN, such as eating, weight and shape concerns (Table 6).

**TABLE 6:** Characteristics and main results of studies (RCT, NRCT, UT) conducted on effects of combined physical exercise interventions in patients with AN.

References	Study type	Type of therapy	Participants’ characteristics Sample size (% female), mean age (SD) Mean BMI (SD) and ED type (%).	Post-intervention dropout, n (%)	Follow-up dropout, n (%)	Treatment settings	Intervention parameters		Outcomes
		ET + SC	n = 8 (100%), 29 (± 4.4) 20.26 (± 1.8), AN (100%)	3 (37.5%)	/		Stretching, resistance and aerobic exercise	D = NR I = progressive intensity (7 levels) F = 3x/week P = 3 months NA = no	
Thien et al. (61)	RCT					Outpatient			**Physical health:** BMI; %BF; **Mental health:** SF-36.
		SC	n = 8 (87.5%), 36.1 (± 7.9) 17.2 (± 1.6), AN (100%)	1 (12.5%)	/		Individualized: Yes I/P ratio = NR		

		ET + SC	n = 112 (100%), 20.04 (± 5.7) 14.98 (± 1.96), AN (80%), EDNOS (20%)	24 (21%)	15 (13%)		CBT coupled with PE (yoga, recreational activity, body exploration, dual task exercises)	D = 100 min I = NR F = 2x/week P = 4 weeks NA = no	**ED symptoms:** EDE-Q. **Physical health:** BMI; **Mental health:** CES*; CET*; BDI-II; BSI; DERS.
Dittmer et al. (62)	RCT					Inpatient			
		SC	n = 95 (100%), 18.32 (± 5.19) 15.35 (± 1.86), AN (68%), EDNOS (22%)	21 (22%)	19 (20%)		Individualized: yes I/P ratio = 1/1 and NR for group sessions		

		ET + SC	n = 15 (94%), 24.3 ± 3.4 20.3 ± 2.7, AN (33%), BN (60%), OSFED (7%)	3 (20%)	0 (0%)		Educational program and PE (45-60min playful activities, team sports, body-oriented exercises) (group)	D = 120 min I = NR F = 2x/week P = 12 weeks NA = no	**ED symptoms:** E DE-Q; EDI-2 subscales drive for thinness, bulimia and body dissatisfaction. **Physical health:** BMI; Exercise quantity with accelerometer; **Mental health:** CES;
Zeeck et al. (63)	RCT					Outpatient			
		SC	n = 11 (100%), 27.2(± 8.8) 19.2 (± 2.1), AN (45.5%), BN (36.4%), OSFED (18.1%)	0 (0%)	0 (0%)				
							Individualized: yes I/P ratio = 1/1 and 1/5-8		CET*; EDS; SCL-27; BDI-II; IPAQ;

		ET + SC	n = 19 (100%), 15.94 (± 2.45) 14.82 (± 1.01), AN (100%)	NR	2 (10%)		stretching, posture enhancement, weight training, social sport and occasional aerobic activity with no impact (individually and group)	D = 180 min I = NR F = NR P = 6 weeks NA = no	**Physical healt h:** BMI; Weight; Weight gain.
Touyz et al. (68)	NRCT					Inpatient			
		SC	n = 20 (100%), 20 (± 5.28) 14.28 (± 1.32)	NR	0 (0%)		Individualized: yes I/P ratio = 1/1 and NR for group sessions		

		ET + SC	n = 127 (100%), 22.49 (± 7.96), 18.45 (± 5.24), AN (50%), BN (33%), EDNOS (17%)	NR	/		stretching, yoga, Pilates, weight training, recreational activity, endurance activity (group)	D = 60 min I = NR F = 4x/week P = length of the stay NA = no	**ED sympto ms:** items from EDE-Q. **Physical health:** BMI; Weight gain*; **Mental health:** OEQ*; OBC-AC; EDPEX.
Calogero& Pedrotty (69)	NRCT					Inpatient			
		SC	n = 127 (100%), 23.14 (± 8.72) 20.54 (± 5.98), AN (41%), BN (37%), EDNOS (22%)	NR	/		Individualized: yes I/P ratio = 1/1 and NR for group sessions		

		ET + SC	n = 18 (89%), 24.8 (± 3.5) 21.7 (± 4.1), AN (33%), BN (56%), EDNOS (11%)		/		Educational program and PE (45-60’ playful activities, team sports, body- oriented exercises) (group)	D = 120 min I = NR F = 2x/week P = 12 weeks NA = no	**ED sympt oms:** EDI-2 subscales drive for thinness and body dissatisfaction; EDE-Q. **Physical health:** BMI; **Mental health:** CES*; SF-12.
Schleg el et al. (70)	NRCT			11 (34%)		Outpatient			
		SC	n = 18 (94%), 26.1 (± 6.8) 21.3 (± 5.3), AN (33%), BN (56%), EDNOS (11%)		/		Individualized: yes 1/1 and NR for group sessions		

							CBT coupled with PE (yoga, recreational activity, body exploration, dual task exercises)	D = 100 min I = NR F = 2x/week P = 4 weeks NA = no	**ED symptoms:** EDI-2*. **Physical health:** BMI*; **Mental health:** CES*; CET*; BDI-II*; BSI*; ERSQ*.
Dittmer et al. (74)	UT	ET + SC	n = 32 (100%), 22.6 (± 8.25) 15.67 (± 1.54), AN (81%), BN (6%), EDNOS (13%)	9 (28%)	/	Inpatient			
							Individualized: yes 1/1 and NR for group sessions		

							Resistance exercise and shadow boxing	D = 90 min I = low F = 1x/week P = 8 weeks NA = no	**ED symptoms:** EDE-Q*. **Physical health:** BMI*; **Mental health:** GLTEQ*; EDSR*; EDQ*; DUKE-HP*.
Kern et al. (75)	UT	ET + SC	n = 41 (100%), 16.35 (± 1.33) 16.76 (± 2.03), AN (100%)	12 (29%)	/	Inpatient	Individualized: yes 1/1 and NR for group sessions		

SD, standard deviation; ED, eating disorders; ET, exercise therapy; BMI, body mass index; ED, eating disorder; RCT, randomized controlled trial; NRCT, non-randomized controlled trial; UT, uncontrolled trial; SC, standard care; NR, not reported; AN, anorexia nervosa; BN, bulimia nervosa; EDNOS, eating disorder not otherwise specified; OSFED, other specified feeding or eating disorder; I/P ratio, Instructor/Patient ratio; D, session duration; I, intensity; F, frequency; P, period; NA, nutritional adjustment;%BF,%body fat; SF-36,medical outcomes study 36-items short-form health survey; EDE-Q, eating disorder examination questionnaire; OEQ, obligatory exercise questionnaire; OBC-AC, objectified body consciousness scale—appearance control subscale; EDPEX, eating disorder patient’s expectations and experiences of treatment questionnaire; CES, commitment exercise scale; EDI-2, eating disorder inventory 2; CBT, cognitive behavioral therapy; PE, physical exercise; CET, compulsive exercise test; BDI-II, Beck depression inventory-II; BSI, brief symptom inventory; ERSQ, emotion regulation skills questionnaire; DERS, difficulties in emotion regulation scale; GLTEQ, Godin leisure-time exercise questionnaire; EDSR, exercise dependence scale-revised; EDQ, exercise dependence questionnaire; DUKE-HP, DUKE health profile; EDS, exercise dependence scale; SCL-27, symptom check list-27; IPAQ, international physical activity questionnaire; *, significant improvement for the intervention group.

### Quality assessment of the studies

Overall, according to the PEDro scale criteria, the 27 studies presented a fair quality with a mean score of 4.29 (± 1.66), ranging from 2 to 7 on a scale of 0 to 10 (Table 7). The PEDro mean score was slightly higher when only RCTs were considered (i.e., 5.61 ± 0.96), but still reflected a fair quality of the methodological procedures. When only NRCTs were considered, mean score was 3.86 (± 0.90) which was between fair and poor quality. When only UTs were considered, mean score was 2.28 (± 0.75) which revealed a poor quality. Among the 27 studies, seven studies (26%) were considered to be of good quality (score = 6–8), twelve studies (44%) were considered to be of fair quality (score = 4–5), and eight (30%) studies were considered to be of poor quality (score ≤ 3). The only study that examined the effect of aerobic exercise alone in patients with AN had a score of 4, indicating a fair methodological quality for this study. Concerning the studies including resistance exercises (RCTs), the mean score was 5.71 (± 1.11) which revealed good quality. The 11 studies based on MBPE (3 RCT, 4 NRCT and 4 UT) obtained a mean score of 3.63 (± 1.75) indicating fair quality. When only RCTs were considered for MBPE studies, the PEDro mean score was 6 (± 0), indicating good methodological quality. The mean score obtained for the eight studies based on combined PE intervention (3 RCT, 3 NRCT and 2 UT) was 4 (± 1.41), or fair quality. Regarding combined PE, when only RCTs were considered, the PEDro mean score was 5 (± 1), reflecting the same level of quality.

**TABLE 7:** PEDro scale scores.

		Random assignment	Concealed allocation	Baseline group similarity	Blind subjects	Blind therapists	Blind assessors	Less than 15% dropout	Intention-to-treat analysis	Between-group comparisons	Estimating the effect and its variability	Total
*A. Studies conducted on effects of aerobic exercise in patients with AN*												

1	Tokumura et al. (64)*	0	0	1	0	0	0	0	1	1	1	4

	*Mean score RCT* = *0/10; Mean of NRCTs scores* = *4/10; Mean of UTs scores = 0/10*										*Mean score (A)*	4

*B. Studies conducted on effects of resistance exercise in patients with AN*												

1	Szabo & Green (49)**	1	0	1	0	0	0	0	1	1	1	4

2	Chantler et al. (50)**	1	0	1	0	0	0	0	1	1	1	5

3	Fernandez-del-Valle et al. (56)**	1	1	1	0	0	0	1	1	1	1	7

4	Fernandez-del-Valle et al. (51)**	1	0	1	0	0	0	1	1	1	1	6

5	Fernandez-del-Valle et al. (52)**	1	0	1	0	0	1	1	1	1	1	7

6	Fernandez-del-Valle et al. (53)**	1	0	1	0	0	0	0	1	1	1	5

7	Martin et al. (57)**	1	0	1	0	0	0	1	1	1	1	6

	*Mean score RCT* = *5,71/10; Mean of NRCTs scores* = *0/10; Mean of UTs scores = 0/10*										*Mean score (B)*	*5, 71*

*C. Studies conducted on effects of mind-body physical exercise interventions in patients with AN*												

1	Cook-Cottone et al. (71)	0	0	0	0	0	0	0	1	0	1	2

2	Carei et al. (58)**	1	0	1	0	0	0	1	1	1	1	6

3	Moscone et al. (8)*	0	0	0	0	0	0	1	1	1	1	4

4	Catalan-Matamoros et al. (59)**	1	1	1	0	0	0	0	1	1	1	6

5	Hall et al. (72)	0	0	0	0	0	0	0	1	0	1	2

6	Pacanowski et al. (60)**	1	0	1	0	0	0	1	1	1	1	6

7	Keizer et al. (65)*	0	0	0	0	0	0	1	1	0	0	2

8	Diers et al. (73)	0	0	0	0	0	0	0	1	0	1	2

9	Martinez-Sanchez et al. (54, 55)	0	0	0	0	0	0	0	1	0	1	2

10	Savidaki et al. (66)*	0	0	0	0	0	0	1	1	1	1	4

11	Demartini et al. (67)*	0	0	0	0	0	0	1	1	1	1	4

	*Mean score RCT* = *6/10; Mean of NRCTs scores* = *3.50/10; Mean of UTs scores = 2/10*										*Mean score (C)*	*3, 63*

*D. Studies conducted on effects of combined physical exercise interventions in patients with AN*												

1	Touyz et al. (68)*	0	0	0	0	0	0	1	1	1	1	4

2	Thien et al. (61)**	1	0	0	0	0	0	0	1	1	1	4

3	Calogero & Pedrotty (69)*	0	0	1	0	0	1	0	1	1	1	5

4	Schlegel et al. (70)*	0	0	1	0	0	0	0	1	1	1	4

5	Dittmer et al. (74)	0	0	0	0	0	0	0	1	0	1	2

6	Dittmer et al. (62)**	1	0	1	0	0	0	0	1	1	1	5

7	Kern et al. (75)	0	0	0	0	0	0	0	1	0	1	2

8	Zeeck et al. (63)**	1	0	1	0	0	0	1	1	1	1	6

	*Mean score RCT = 5/10; Mean of all NRCTs scores = 3.86/10; Mean of all UTs scores = 2/10*										*Mean score (D)*	4

	*Mean of all RCTs scores = 5.61/10; Mean of all NRCTs scores = 3.87/10; Mean of all UTs scores = 2/10*										*Mean of all scores*	4, 29

**Nandomized controlled trial; *non-randomized controlled trial; PEDro score 9-10 = high quality; PEDro score 6-8 = good quality; PEDro score 4-5 = fair quality; PEDro score ≤ 3 = poor quality.

## Discussion

This systematic review reveals that ET supervised by exercise professionals does not adversely affect the health of patients with AN, either in outpatient or inpatient settings; therefore, PE cannot be systematically contraindicated. Furthermore, beneficial effects have been identified both on the symptomatology of AN and on physical and mental health. In most of the controlled studies presented in this review, patients with ET intervention in addition to their usual care showed similar (49, 58, 61, 67, 68) or even greater improvements (50–53, 56, 57, 59, 60, 62–64, 66, 69, 70) than the control group in all the dimensions assessed. We examined comparable studies for differences in observed effects between inpatients and outpatients and found little or no difference. For example, all studies investigating the impact of combined PE interventions, with the exception of Dittmer et al. (74), found no increase in BMI post-program, regardless of whether patients were hospitalized. In the same way, among these studies, the three that examined the effect of combined PE interventions on compulsive exercise in patients with AN using the CET questionnaire found a significant increase in CET score after the program for inpatients (59) as well as outpatients (60, 73).

The study by Tokumura et al. (64) was, to our knowledge, the only study to investigate the effectiveness of exclusively aerobic exercise on patients with AN, which is unsurprising given that this type of PE is generally avoided in the care of patients with AN. Overly intensive exercise, particularly aerobic exercise modes such as running and swimming, may seem inappropriate for patients with severe undernutrition due to its high energy expenditure requirement, which could lead to even greater weight loss or other medical risks (85). Nevertheless, their results revealed increase in cardiorespiratory capacity and BMI. Thus, this specific cardiorespiratory training program of 30 minutes of stationary bicycling at the anaerobic threshold five times per week appears to be beneficial for patients with AN.

In most studies that included resistance exercise program, interventions parameters were from low-to-high intensity, 2-3 times per week for at least 8 weeks. They revealed significant increases in muscle strength, no adverse effect on bone density and a faster stabilization of vital signs. The four studies conducted by Fernandez-del-Valle et al. (51–53, 56) made an important contribution to this topic. Their results first revealed that resistance exercise was not harmful to patients’ health (48) and further showed that higher intensity and frequency contributed to enhanced effects on strength and anthropometric parameters (51–53). The findings of the studies assessing resistance exercise interventions suggest that this type of PE is suitable for patients with AN to improve muscle strength and size (50–53, 56), and restore bone mineralization (57). Finally, it is important to mention that in all of these studies, additional caloric intake was given to the intervention group to counterbalance the energy expenditure, except for the study of Fernandez-del-Valle et al. (56) and the study of Martin et al. (57).

Almost half of the studies that included MBPE showed significant improvements in specific symptoms of AN and psychological associated disorders, such as body shape and body concerns, body dissatisfaction, depression, anxiety and quality of life (54, 59, 60, 66, 71–73). MBPE such as yoga or Pilates offers patients the opportunity to be physically active while avoiding weight loss and excessive caloric expenditure (33). In addition, the mobilization of an often-rejected body leads patients to develop more positive feelings about their body image and a healthier relationship with their body (8). One study also revealed the positive impact of MBPE on the sleep quality and sleep efficiency of patients (55). All of these results tend to provide evidence of the effectiveness of this type of PE in patients with AN. However, they must be taken with caution because most of these studies are NRCTs or UTs, only three RCTs were carried out (58–60) and two showed significant improvements (59, 60) (i.e., ED symptoms, quality of life, body attitude and negative affects).

Finally, most studies that examined the effects of ET intervention using combined PE showed positive effects in patients with AN, especially on dysfunctional exercise behaviors, with a reduction of compulsive exercise and exercise dependence (62, 63, 69, 70, 74, 75). In addition, a few studies revealed that combined PE contributed to the increase of weight (69, 74, 75), and reduction in specific symptoms of AN (74, 75), as well as anxiety and depression (74). These findings are congruent with the primary goals of AN treatment. Therefore, ET intervention including various types of PE seems promising for clinical practice and future research. However, once again, results must be taken we caution as there were only three RCTs (61–63) and only two revealed significant improvements (62, 63) (i.e., compulsive exercise and commitment to exercise). It is worth to mention that other than BMI, physical and biological parameters such as muscle strength, muscle endurance, respiratory capacities, and blood analysis have never been assessed for this type of PE intervention in AN patients.

Taken together, all these results suggest that, despite the heterogeneity of the results (especially regarding MBPE), ET may have multiple benefits for patients with AN, which depend on the type of PE practiced. Therefore, it seems difficult to limit recommendations for PE within clinical management to a single type of PE, unless one type of PE is targeted according to the primary therapeutic goals. For example, if the main therapeutic goal is to improve the patient’s muscle mass and strength, resistance training could be the best choice according to data from the literature. Indeed, the majority of studies that examined the effects of a resistance training protocol in anorexic patients have shown significant gain in muscle mass and strength in these patients. However, as studies that examined the benefits of other types of PE in patients with AN have not measured effects on muscle function, it is not possible to state that other types of PE are not equally effective in improving muscle mass and strength. Thus, even if this systematic review could be useful for clinicians to make informed choices about which type of PE to recommend, further studies are needed to explore and compare the effects of each type of PE on the same health outcomes.

Moreover, it can be assumed that the benefits achieved may also vary depending on the ET intervention parameters. Results of the present review support the argument that for any given PE, some specific parameters such as the period or frequency of PE sessions could be more effective than others in reducing the symptoms of AN, or in improving the physical and psychological health of patients. However, it is difficult to make precise recommendations regarding specific ET intervention parameters to improve the therapeutic strategy of AN treatment. Indeed, results revealed that the duration, as well as the frequency and length of the sessions, varied greatly between different types of PE and within similar intervention types. In ET intervention including resistance exercise, patients were commonly asked to engage in an 8-week program, with two or three 60-minute sessions per week. Concerning MBPE intervention, the frequency of sessions usually varied from one to two per week, for 8–12 weeks, with sessions ranging from 60 to 120 minutes. It is in the ET intervention including combined PE that can be observed the higher heterogeneity in parameters, with a period of four to twelve weeks, a frequency ranging from one to four per week and length varying from for 60–180 minutes per session.

As a consequence and in agreement with the collective expertise report on the health effects of PE in patients with chronic diseases, coordinated by the French National Institute of Health and Medical Research (86), the major public health issue is now to determine the most efficient ET intervention parameters and to adapt them to the patients’ individual needs.

## Limitations and future directions

In recent years, there has been an increasing interest in clinical research focusing on the feasibility and effectiveness of ET intervention as a therapeutic strategy for patients with AN, or more broadly for patients with ED. Indeed, most of the studies included in the present review (67%) were published in the last decade. However, this systematic review reveals that the quality of the studies remains too small to obtain a good level of evidence. Indeed, the overall quality of the studies included, measured using the PEDro scale, was fair with a total mean score of 4.29 (± 1.66). The most common methodological weaknesses were the absence of randomization, the lack of concealed allocations, the non-blinding of therapists and assessors, and a high dropout rate. However, in the case of PE interventional research, given the near impossibility of blinding subjects (87), it was not surprising that all studies scored 0 for this criterion.

Moreover, we identified some methodological limitations that should be taken into consideration in the interpretation of the findings. First, only 13 of the 27 studies were RCTs, which may partly explain a low overall score on the PEDro scale. In addition, 13 of the 27 studies were pilot studies. Regarding the number of RCT conducted for each type of PE, the level of evidence was different with a higher level for resistance exercise (i.e., 7 RCT) and a lower level for MBPE (i.e., 3 RCT, 4 NRCT, and 4 UT), combined PE (i.e., 3 RCT, 3 NRCT, 2 UT) and aerobic exercise (i.e., 1 NRCT). Further, most of the studies did not report sample size calculation and other major statistical lacks can be reported: 16 studies did not report a primary outcome while some studies reported multiple outcomes, but no formal adjustments were made for multiple comparisons. Only two studies performed a Bonferroni correction for multiple comparisons (51, 53). These different limitations underscore the need for caution in interpreting the results, particularly for studies without a control group, and should be addressed in future studies to provide greater methodological rigor and greater confidence in the research findings.

Other methodological limitations should be pointed out, such as the vast diversity of tools used to measure identical variables. This was particularly true for the measurement of body composition, which differed from one study to another. For example, while in studies by Fernandez-del-Valle’s team (51–53), body composition was assessed by BMI calculation coupled with a measure of fat mass (FM), in the study by Martinez-Sanchez et al. (55), it was assessed by using a bioelectrical impedance analysis. Other studies simply assessed body composition using BMI calculation. However, it is now accepted that BMI, although a quick way to assess if a patient is underweight or overweight, remains an imprecise measure of body composition and actual metabolic risk, as its calculation considers weight as a whole and does not distinguish between fat and lean mass. For a more accurate and reliable measurement of body composition, some authors suggest the use of bioelectrical impedance analysis or dual x-ray absorptiometry (DEXA) measurement (88). Therefore, more direct assessments of body composition are needed in future research. In addition, the present review highlighted a diversity of measures used to assess the specific symptoms of AN (or ED) and their level of severity, which limits interpretation of the results and comparisons among studies. Thus, seven of the studies used the eating disorder examination questionnaire (EDE-Q), five used the eating disorder inventory 2 (EDI-2), two used the eating disorder inventory 1 (EDI), one used the eating disorder inventory 3 (EDI-3), one used the eating attitude test 40 (EAT-40), and one used the eating attitude test 26 (EAT-26). The same heterogeneity can be reported for other outcomes (for example, quality of life, exercise dependence, body dissatisfaction, PE time).

It is also important to note that the studies included very few men, with a total of 16 men out of the 27 studies included, compared to 1300 women. This small sample can be explained by the ratio usually reported in AN with only 1 man for 10 women. It can also raise the question of whether the findings of this systematic review apply to male participants regarding their low representativeness. Hence, a study including only male participants would be a first and major contribution to this field.

Although the main ET intervention parameters were usually reported (i.e., session duration, frequency and period), individualization, instructor to patient ratios, exercise intensity and adjustment of caloric intake were not always indicated, as well as the content of ET intervention was poorly detailed in most of the studies. Indeed, only a few studies mentioned the level of exercise intensity and only six detailed how progression was managed through the program (51–53, 56, 61, 75). According to these authors, the intensity of PE for patients with AN should be systematically controlled and graded. Indeed, a lack of gradation may result in anorexic patients exercising at a very high intensity, and therefore lead to further weight loss. In addition, this review showed that only a few authors described the delivery method (71) and the structure of the program (54, 55, 60, 61, 69, 71). Overall, the ET interventions were poorly described in the majority of studies, which makes reproducibility and comparability difficult.

Another bias can be identified. Indeed, among the studies, four included ET intervention coupled with another type of therapy (educational program or cognitive behavioral therapy) and did not allow to isolate the effects of ET intervention, which alone cannot completely explain the improvements observed in patients. Moreover, especially for studies conducted with inpatients (i.e., 10 studies among all selected studies), it is common for patients to receive other therapies in addition to ET intervention as part of their protocol of care (e.g., psychotherapy, nutritional follow-up). These treatments, which are rarely mentioned in studies, may also play a role in improving patients’ health and may represent a bias in the results. Finally, it is unfortunate that in studies that included a majority of AN patients among other ED, results were not extracted, presented and analyzed separately for AN. Indeed, it would be interesting to have access to detailed results by type of ED, in order to examine the differences in effect according to pathology and to better target potential clinical applications.

## Conclusion

This systematic review provides an overview of existing evidence regarding the effect of ET intervention in patients with AN. Specific benefits have been emphasized according to the type of PE intervention and can be considered for future research or clinical implications. Nevertheless, this review does not allow us to affirm that the effects obtained are related exclusively to a type of PE. For example, resistance training exercise revealed significant increase in muscular strength, which could also be achieved through the practice of a yoga or Pilates program. To our knowledge, this has not been examined in any previous study.

In addition, this review highlights several limitations of the existing literature, such as inconsistent results, a fair methodological quality or heterogeneity of measures, which greatly contribute to lowering the quality of evidence of the studies and make it difficult to establish specific recommendations for patients with AN. However, ET intervention seems to be emerging as a therapeutic strategy that can contribute to the well-being and the recovery of patients with AN and does not induce deleterious effects as long as it is adapted to patients’ profiles and supervised by exercise professionals. Further work is needed in this field of research to determine whether, in addition to being accepted and not limited, the integration of ET intervention within the management of patients with AN is truly effective, on what health outcomes and to what extent.

## Data availability statement

The original contributions presented in this study are included in the article/supplementary material, further inquiries can be directed to the corresponding author/s.

## Author contributions

MT, PL, and AG planned the research. MT and PL conducted the review. AG checked the search strategy and results. All authors contributed to write and read the manuscript, and approved the manuscript.
